# RETRACTED: Akter et al. Potential Role of Natural Products to Combat Radiotherapy and Their Future Perspectives. *Molecules* 2021, *26*, 5997

**DOI:** 10.3390/molecules28248091

**Published:** 2023-12-14

**Authors:** Rokeya Akter, Agnieszka Najda, Md. Habibur Rahman, Muddaser Shah, Sylwia Wesołowska, Syed Shams ul Hassan, Sidra Mubin, Parveen Bibi, Saeeda Saeeda

**Affiliations:** 1 Department of Pharmacy, Jagannath University, Dhaka 1100, Bangladesh; rokeyahabib94@gmail.com; 2 Department of Global Medical Science, Wonju College of Medicine, Yonsei University, Wonju 26426, Gangwon-do, Republic of Korea; 3 Department of Vegetable and Herbal Crops, University of Life Sciences in Lublin, 50A Doświadczalna Street, 20-280 Lublin, Poland; 4 Department of Pharmacy, Southeast University, Banani Street, Dhaka 1213, Bangladesh; 5 Department of Botany, Abdul Wali Khan University Mardan, Mardan 23200, Pakistan; parveentariq825@gmail.com (P.B.); syeeda177@gmail.com (S.S.); 6 Natural and Medical Sciences Research Center, University of Nizwa, Nizwa 616, Oman; 7 Institute of Soil Science and Environment Shaping, University of Life Sciences in Lublin, 7 Leszczyńskiego Street, 20-069 Lublin, Poland; 8 Shanghai Key Laboratory for Molecular Engineering of Chiral Drugs, School of Pharmacy, Shanghai Jiao Tong University, Shanghai 200240, China; shams1327@yahoo.com; 9 Department of Botany, Hazara University Mansehra, Mansehra 21310, Pakistan; shahhu123@gmail.com

This published review [1] has been retracted due to its significant overlap with a previously published review [2].

Following publication, the editorial office was contacted regarding the overlap.

Adhering to the complaint’s procedure, the editorial office conducted an investigation that confirmed the similarities. The Editorial Board confirmed that the overlap of the topics, compounds and figures was significant, and that the review should be retracted.

This retraction was approved by the Editor-in-Chief of the journal *Molecules*. 

The authors agree with this retraction. 
